# A controlled trial of Cognitive Behavioural Therapy-based strategies for insomnia among in-school adolescents in southern Nigeria

**DOI:** 10.1186/s13034-021-00406-1

**Published:** 2021-09-25

**Authors:** Diseyei R. Egbegi, Tolulope Bella-Awusah, Olayinka Omigbodun, Cornelius Ani

**Affiliations:** 1 Centre for Child and Adolescent Mental Health, University of Ibadan, Ibadan, Nigeria; 2 Department of Psychiatry, College of Medicine, University of Ibadan, Ibadan, Nigeria; 3 Division of Psychiatry, Imperial College London, Hammersmith Hospital Campus, 2nd Floor Commonwealth Building, Du Cane Road, London, W12 0NN UK; 4 Surrey and Borders Partnership, NHS Foundation Trust, Leatherhead, Surrey, UK

**Keywords:** Insomnia, Clinical Trial, CBT, CBT-I, Adolescents, Nigeria

## Abstract

**Background:**

Sleep difficulties are highly prevalent among adolescents, and are associated with significant impairments. The effectiveness and acceptability of Cognitive Behavioural Therapy-based (CBT-based) treatment for insomnia in adolescents is established for High Income Countries, but unknown for African settings. Thus, the aim of this study was to assess the effect of CBT-based intervention among in-school adolescents with sleep difficulties in Southern Nigeria.

**Methods:**

This was a pilot controlled trial involving 50 adolescents with highest ranked scores on the Insomnia Severity Index (ISI) recruited from four schools (two government and two privately owned). Balloting was used to assign two schools (public and private) with 25 participants to the intervention group, and the other two schools (public and private) with 25 participants as waiting-list controls. The two groups were dyad-matched for baseline ISI scores, gender, and type of school to reduce baseline differences. The treatment group received weekly group-based manualised CBT-based intervention over 5 weeks. Primary outcome was ISI score at 6th week. Secondary outcomes were sleep onset latency (SOL), Total sleep duration (TSD), depressive symptoms, sleep hygiene, and knowledge about sleep.

**Results:**

Participants were aged 13–17 years (M = 14.9, SD = 1.16) and consisted of 18 males and 32 females. Controlling for baseline scores, the intervention group showed significantly lower post-intervention insomnia scores compared with the control group {F (1, 34) = 1.10, p = 0.0001, (ηp^2^ = 0.59}, shorter SOL {F (1, 33) = 1.41, p = 0.0001, ηp^2^ = 0.39}, longer TSD {F (1, 33) = 1.03, p = 0.0001, ηp^2^ = 0.47}, lower depressive symptoms {F (1, 31) = 1.32, p = 0.002 (ηp^2^ = 0.34}, higher knowledge of sleep {F (1, 34) = 1.02, p = 0.001, ηp^2^ = 0.36}, but no significant change in sleep hygiene {F (1, 32) = 1.08, p = 0.08, ηp^2^ = 0.15}. All participants in the intervention group rated the programme as good or excellent.

**Conclusion:**

This pilot CBT-based intervention for adolescents with insomnia was feasible, well received and showed promising efficacy in this setting. Larger controlled trials are recommended to establish the generalisability of these findings in this region.

*Trial registration* Pan African Clinical Trial Registry (Registration Number PACTR202001710494962)

## Introduction

Sleep difficulties are highly prevalent among adolescents in all regions of the world [1]. Poor sleep in this age group is associated with significant impairments including, poor concentration, poor emotional regulation, depression, anxiety, and poor academic performance [2]. Sleep difficulties can worsen other underlying mental disorders such as Attention Deficit and Hyperactivity Disorder (ADHD) [3]. Impaired attention due to disrupted sleep increases the risk of accidents with the potential for fatal outcomes [2]. Left untreated, sleep difficulties in adolescence tend to become chronic, thereby prolonging the impairments [2].

The high prevalence and serious consequences of sleep difficulties among adolescents make early recognition and treatment highly imperative [4]. Systematic reviews and meta-analysis support the effectiveness of psychological interventions for adolescent insomnia such as Cognitive Behavioural Therapy for insomnia (CBT-I) [4–6]. However, despite the good evidence-base for interventions like CBT-I, most affected adolescents go untreated due to limited access to the intervention in most countries [7]. This treatment gap is likely to be worse in resource-limited regions in Low and Middle Income Countries (LMICs) like Nigeria.

While studies of CBT-based interventions for insomnia have started to emerge among adult populations in Africa [8], this is not yet the case for children and adolescents. For example, a recent systematic review of broader modalities of psychological intervention for paediatric insomnia cited studies from mostly High Income Countries (HICs) but featured no studies from Africa [9]. Thus, while the evidence-base for psychological interventions for adolescent insomnia is good in HICs, there remains absence of well-established, effective and or culturally appropriate psychological interventions for adolescents in resource-limited settings in Africa. The huge multidimensional socio-ecological differences between HICs and LMICs mean that even if a psychological intervention has evidence-base in HICs, it is likely to require adaptation in LMICs to be effective and accessible [10, 11]. A key relevant context in LMICs is severe shortage of highly skilled mental health professionals such that countries like Nigeria have 1.4 mental health workers per 100,000 population compared with a global average of 9.0 per 100,000 [12]. This huge human resources gap places in question the viability of interventions that rely on wide-spread delivery by highly skilled professionals.

Furthermore, there are several contextual differences between HICs and LMICs like Nigeria that are specifically relevant to psychological interventions for insomnia such as CBT-I. For example, “stimulus control” is a key component of CBT-I which recommends not using the bedroom for activities not related to sleep [13, 14]. However, this advice may be impractical in the overcrowded living conditions common in LMICs like Nigeria whereby many adolescents have little option than to use their bedroom for academic activities due to shortage of living spaces. A similar impracticality may apply to the CBT-I strategy that aims to reduce time spent awake in bed by encouraging the person who is unable to sleep to get out of bed and go to another room for a period [13]. In the context of Nigeria, the adolescent may not have another room to go to, or to do so could interfere with the sleep of other family members with whom they may be sharing the same sleeping space. Thus an otherwise effective intervention like CBT-I requires more nuanced application in the context of LMICs like Nigeria because uncontextualised application could unwittingly trigger family difficulties. In summary, without appropriate contextualisation, typical packages of psychological interventions for adolescent insomnia that are effective in HICs may not be feasible or applicable in LMICs like Nigeria due to lack of specialist expertise to deliver the intervention and or poor match between the treatment strategies and the socio-ecological realities in this setting.

Thus, the objective of this pilot study is to evaluate a locally adapted course of psychological intervention for adolescent insomnia that draws on components of CBT-I that we judged to be feasible and deliverable in this context. The adaptations included emphasis on the following CBT-I components; psychoeducation, sleep hygiene, restriction of daytime naps, relaxation techniques, and a simple cognitive strategy for managing bedtime worries. Further adaptations included; shortening the course of the intervention to 5-sessions—to improve feasibility, delivering it in groups—to improve cost-effectiveness and feasibility, delivering it in schools—to improve access, and delivery by a professional with no prior specialist certification in CBT-I. The latter point aims to explore the feasibility of delivery by a wider range of potential facilitators if the intervention is found to be effective. The study also explored additional parameters to inform future definitive trials such as viability of recruitment strategies and reliability of outcome measures. This study responded to the call for more diversity and better inclusivity [15] in the global literature on psychological interventions for young people with sleep difficulties.

## Method

### Study design and location

This was a parallel two-group intervention study conducted in four secondary schools in Yenagoa, Bayelsa State in Southern Nigeria. Two of the 35 government-funded secondary schools in the area were purposively selected based on closest match on student population, staff-student ratio, and neighbourhood type. A similar procedure was used to select two of the 117 privately owned secondary schools. Balloting was used to assign one government owned and one privately owned schools as “intervention sites” and the other two schools as waiting-list controls. Ethical approval was given by the Bayelsa State Health Research Ethics Committee (BSHREC/Vol. 1/19/10). The study was registered with the Pan African Clinical Trial Registry (Registration Number PACTR202001710494962).

### Participants

**Fig. 1:**
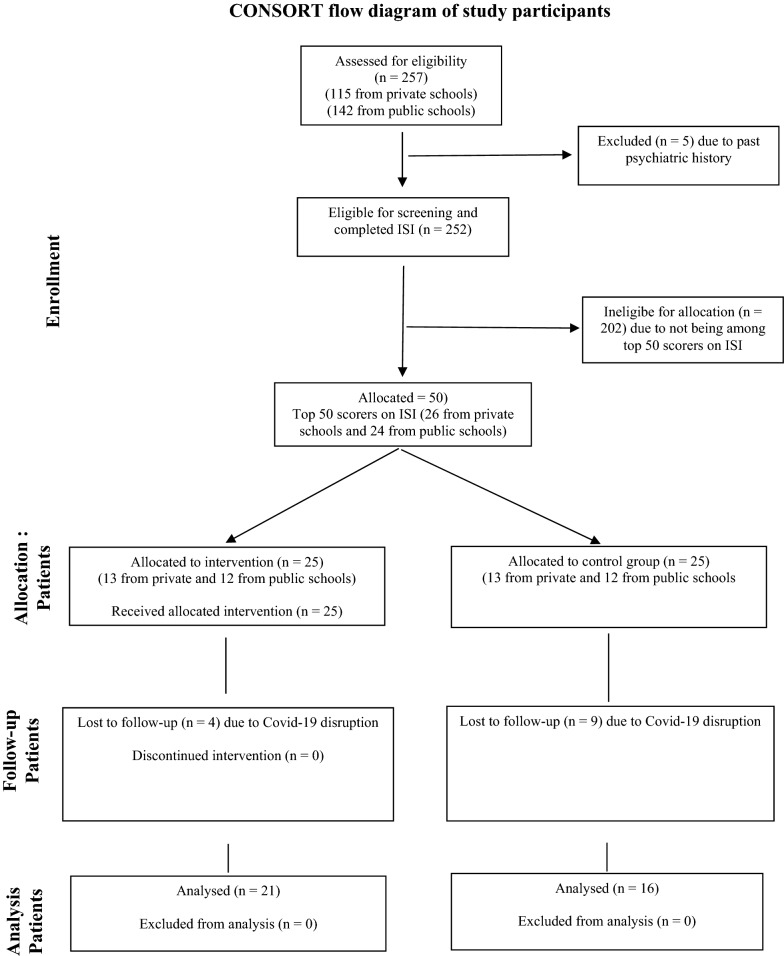
CONSORT flow diagram of study participants

Participants were in-school adolescents aged 13–17 years whose parents or carers provided consent and who themselves gave assent. A total of 257 students were selected by computer-generated random numbers from the four study schools and invited to take part in the study. After excluding five students with a history of psychiatric disorder, 252 students completed the Insomnia Severity Index (ISI) [16]. The top 25 students with consecutively highest ISI scores in the two intervention schools were selected and dyadically matched with 25 students from the control schools on ISI scores, gender, and type of school. Prior sample size calculation identified that a minimum of 16 participants in each group was adequate to detect a reduction of one standard deviation in ISI scores in the intervention group compared with the control group with 80% power and 5% two-sided alpha [17]. This figure was increased to 25 in each group to account for potential attrition. Previous pilot intervention studies in this region have shown that similar sized samples were adequate to identify the level of difference hypothesised in this study [18, 19]. In addition to lack of consent, students were to be excluded if they had a history of psychiatric disorder or their teachers identified them as having learning difficulties. Only five exclusions occurred and all were due to a history of psychiatric disorder. Twenty one students in the intervention group and 16 controls completed the post intervention outcome measures. All losses to follow-up were due to subsequent Covid-19-related disruptions. Recruitment, intervention and follow-up took place between January and March 2020. Participants’ flow is shown in Fig. 1.

### Procedure

#### Intervention

This was a manualised intervention based on Cognitive Behavioural Therapy strategies for insomnia. The treatment manual was developed by the first author (DRE) under the supervision of the last author (CCA) who is experienced in developing and adapting intervention manuals for use in LMICs [18–22]. The content of the manual was informed by previous seminal works on CBT-I [13, 23] and a recent trial of CBT-I among adolescents [14].

We made four contextual adaptations. First, written tasks such as sleep logs were avoided because our pre-study scoping exercise and prior research experiences in the setting suggested that this would increase the burden of participation, and potentially undermine study feasibility. As a pilot study, we placed a high premium on feasibility. Secondly, we nuanced some components of CBT-I such as the aspect of “stimulus control” that discourages use of the bedroom for non-sleep related activities. In this circumstance, such practice was not discouraged if, for example, the participants needed to do schoolwork in their bedrooms due to shortage of living space. Third, participants were not encouraged to get out of bed and go into another room if they could not fall asleep within 15–20 min, if this did not fit their family’s living circumstances. This is because lack of parental permission and limited living spaces made such practice impractical for most participants. Instead, such participants were encouraged to stay in bed and practice more relaxation techniques, which do not interfere with other members of the household. Fourth, the discussion of “sleep restriction” was limited to avoiding long daytime nap because it reduces sleep drive at bedtime. We did not include detailed advice on bedtime sleep restriction partly because that requires analysis of sleep logs, which were not collected as part of the study as explained earlier. Also the practice of bedtime sleep restriction was not likely to have fitted within most of the participant’s family’s living arrangements.

The manual was designed for group-based delivery to enhance the cost-effectiveness. The intervention was delivered by the first author who holds a degree in Counselling Psychology, but no prior specific certification in CBT-I. The other authors who are experienced in CBT-based interventions in the setting [18, 19, 24] provided weekly supervision. The intervention consisted of weekly sessions of 45 min over 5 weeks delivered in two groups of 12 or 13 students in their own schools. The sessions were conducted during school-breaks in order to limit interference with the students’ academic work. Pre-study scoping exercise suggested that parents were unlikely to consent if the study interfered with the participants’ in-school or after-school academic activities. The sessions were conducted in English because this is the language of instruction in the schools, and the trainer and all the students were fluent speakers. The control group received no treatment during the study. The plan was to offer them a similar intervention if the treatment was found to be helpful. However, this plan was disrupted by Covid-19 pandemic. As an alternative, a leaflet containing the key points in each session was made available to the control group along with advice to contact the first author Toll-free for individual support.

Details of the session contents are set out in Table 1. In summary, the first session set ground rules, and focused on psycho-education including features of adolescent insomnia and the fact that it is treatable. This session also explored some evidence-based benefits of sleep such as; improved memory, better attention and focus, reduction in stress and risk of depression, and reduction in risk of accidents. The second session continued psychoeducation and covered the basic physiology of sleep such as sleep drive and circadian rhythm, and how these link with sleep hygiene. The importance of melatonin and the suppressing effect of light (such as from electronic gadgets); and the adverse effect of caffeine on sleep drive were discussed. The third session extended the discussion on sleep hygiene, and covered contextually relevant aspects of stimulus control and sleep restriction. The fourth session covered the following bedtime relaxation techniques; deep slow breathing, progressive muscle relaxation, and positive imagery. A simple cognitive strategy for managing bedtime worries and anxious ruminations (count your blessings) was included. This cognitive intervention derives from “Positive CBT” [25] and Religious CBT” [26]. Participants were taught how to use gratitude exercises to generate positive affirmations and positive self-talk which are used to challenge, and deny mental space to anxious thoughts. This helps to disengage from negative loops of anxious bedtime rumination that can stop the individual from going to sleep. We have successfully used “count your blessings” as an effective cognitive intervention in a previous trial in the setting [18]. The fifth session revised preceding sessions, and covered relapse prevention. Participants were given brief non-written tasks after each session in order to help them practise the newly learnt skills. Each new session started with a brief review of what was discussed in the previous session and to trouble-shoot specific difficulties experienced while practising the new skills. Of the 25 students in the treatment group, seven attended all five sessions, four attended four sessions, six attended three sessions, one attended two sessions, and one attended a session.

**Table 1:** Session contents

Session	Topic(s)	Content
1	Introduction and setting ground rules Importance of sleep (Psychoeducation-1)	Facilitator and participants introduced each other Group rules agreed including confidentiality Overview of the 5 sessions Discussion of the importance of sleep for good physical health, mental health, and optimum daily function including better concentration and attention and the potential benefits for a student. This was contrasted with the negative consequences of poor sleep Discussed what is adequate amount of sleep for adolescents?
2	Physiology of sleep (Psychoeducation-2)	Review of previous session Discussed how normal sleep occurs including sleep drive (role of Adenosine), and Circadin Rhythm (role of Melatonin) Discussed impact of caffeine on blocking Adenosine and sleep drive, and role of light in blocking Melatonin
3	Sleep hygiene Sleep restriction Stimulus control	Review of previous session Discussed components of sleep hygiene and how they contribute to improving sleep Extended previous discussion on avoiding caffeine after lunchtime due to blocking effect on sleep drive Extended previous discussion on light blocking effect on melatonin and linked it to avoiding electronic gadgets at bedtime and in bed Discussed sleep restriction in terms of avoiding long daytime nap because it reduces bedtime sleep drive Discussed stimulus control as part of improving the sleep environment but acknowledged the impracticality for those who need to do schoolwork in their bedroom
4	Relaxation techniques and managing bed time worries	Reviewed previous session Effect of stress on sleep and how to reduce stress Types of relaxation techniques—deep slow breathing, progressive muscle relaxation, and positive imagery Managing bedtime rumination on worries—by recounting positive experiences in the day and focusing on these to distract from worries “count your blessings” [18] How to make relaxation a habit by practising relaxation techniques throughout the day
5	Revision and relapse prevention	Review of all key messages from sessions 1 to 4 Checking that participants are practising the techniques and troubleshooting any difficulties Relapse Prevention Formal ending of the intervention

### Measures

The primary outcome was reduction in ISI scores at the end of the 6th week. The secondary outcomes were sleep onset latency (SOL), total sleep duration (TSD), depressive symptoms, sleep hygiene, knowledge about sleep, and client satisfaction. All outcome measures were completed at baseline and a week after the last treatment session in one intervention school and both control schools, and immediately after the last session in the second intervention school (shortened due to imminent COVID-19 lockdown). Only the intervention group completed the client satisfaction questionnaire.

#### Sociodemographic questionnaire

This questionnaire sought the personal and family characteristics shown in Table 2. In line with recommendations for measuring socio-economic status (SES) in LMICs [27], and based on previous studies in the region [22, 28], family ownership of the following items; mobile phone, colour TV, refrigerator, computer, motor car, and house was used as a surrogate for SES. Ownership of each item was scored as “1” but ownership of a car was scored as “2” and a house as “4” in order to weight their relatively higher values. The scores were summed to create a “wealth rating”.

**Table 2:** Socio-demographic characteristics of participants in the intervention and control groups

Variables	Intervention group	Control group	t (df)/X^2^	p-value
Age				
Mean (SD)	15.12 (1.13)	14.72 (1.17)	1.23 (48)	0.23
Gender n (%)				
Male	9 (36)	9 (36)	0.00 (1)	1.00
Female	16 (64)	16 (64)		
Marital status of parents n (%)				
Married	16 (64)	15 (60)	0.09 (1)	1.00
Others	9 (36)	10 (40)		
Family type n (%)				
Monogamous	16 (64)	22 (88)		0.10*
Polygamous	9 (36)	3 (12)		
Living with				
Parents	14 (56)	14 (56)		1.00*
Others	11 (44)	11 (44)		
Mother’s education				
Below secondary school	2 (8)	0 (0)		0.72*
Above secondary school	22 (88)	21 (84)		
Father’s education				
Below secondary school	6 (24)	4 (16)		0.49*
Above secondary school	15 (60)	17 (68)		
Wealth Index	6.13 (1.68)	7.54 (1.73)	− 1.43 (48)	0.16

Others category 1 = separated, divorced, one or both parents dead

*Fisher’s exact test

#### Insomnia Severity Index (ISI)

The Insomnia Severity Index [16] is a brief 7-item instrument that assesses the severity, nature and impact of insomnia in the previous 2 weeks. The items are answered on a 5-point Likert scale (0–4) such that scores range from 0 to 28. A score of 15 or above is classified as clinical insomnia, which is further classified as moderate (15–21) or severe (22–28) insomnia. The ISI was analysed as a continuous outcome measure in the current study. The ISI has shown good reliability among adults in Nigeria [29], and showed acceptable internal consistency in the current study (Cronbach’s alpha 0.73). As a pilot study, ascertaining the feasibility of the ISI as a short and reliable measure of insomnia among adolescents in this setting was a specific goal for the current study. The ISI was supplemented with items 2 and 4 in the Pittsburgh Sleep Quality Index [30] to assess SOL and TSD respectively.

#### Short Mood and Feelings Questionnaire (SMFQ)

This is a 13-item self-rated questionnaire for depression in children between the ages of 7–18 years [31]. The items are rated on a three-point Likert scale; “0” (not true), “1” (sometimes), and “2” (true). The instrument has shown good reliability among Nigerian adolescents [32], and the internal consistency in the current study was good (Cronbach’s alpha 0.85).

#### Sleep Hygiene Questionnaire (SHQ)

This consisted of 7 items adapted from the literature on behaviours that adversely impact sleep. It enquired about behaviours in the preceding 2 weeks such as sleeping with a mobile phone in the bedroom, being woken up at night by a mobile phone, staying up at night to use the phone, presence of a TV in the bedroom, watching TV at bedtime, not having a set bedtime, and drinking caffeinated beverages in the evening. The scores were dichotomised with a score of “1” assigned to a “yes” answer and “0” to “no”. The “yes” scores were summed such that higher scores indicate poorer “sleep hygiene”. However, the internal consistency was poor (Kuder–Richardson 20 = 0.50).

#### Knowledge of Sleep Questionnaire (KSQ)

This 7-item questionnaire was based on the psycho-educational component of the intervention manual. Each item was scored as ‘true’, ‘not sure’, or ‘false’. A score of “1” was given for each correct answer and a “0” for “not sure” or an incorrect answer. These dichotomised scores were summed to create a “Knowledge Scale” which showed acceptable internal consistency (Kuder–Richardson 20 = 0.76).

#### Client Satisfaction Questionnaire

Participants in the intervention group rated the programme on a 4-point scale (Excellent, Good, Fair, or Poor), and indicated whether they would recommend it to other adolescents with similar difficulties. They were also asked three open-ended questions to elicit what they liked or did not like about the intervention and their suggestions for improvement [33].

### Statistical analysis

The data was analysed using SPSS Version 20. Per-Protocol analysis of completers was adopted because it was more conservative in the specific circumstance of the study as explained next. Due to Covid-19 disruption, it was not possible to collect post-intervention data from 4 participants in the intervention group and 9 participants in the control group. If Intent to Treat analysis was adopted with imputation of the baseline data to the missing post-intervention data points, this procedure would likely favour the intervention group and increase the risk of Type 1 Error. This is because more post-intervention data losses occurred among the control group that would disproportionately not be mitigated by factors such as regression to the mean. Thus, in this specific circumstance, Per Protocol analysis of completers was more conservative; hence less prone to Type 1 Error.

A series of Kolmogorov–Smirnov and Shapiro-Wilkes tests were used to assess the normality of all continuous measures. Continuous data with p values ≤ 0.05 in the aforementioned tests were log-transformed prior to parametric analyses. Between-group differences in socio-demographic characteristics and outcome measures were examined with the t-test for continuous variables and Chi-square or Fisher’s Exact tests for categorical variables. The treatment effect was determined with Analysis of covariance (ANCOVA) of post-intervention scores on outcome measures while controlling for the respective baseline scores. Effect sizes were calculated as Partial Eta Squared (ηp^2^) with 0.01, 0.06 and 0.14 representing small, medium and large effect sizes.

## Results

### Socio-demographic characteristics of study participants and baseline scores on outcome measures

The participants ranged in age from 13 to 17 years (M = 14.9 years; SD = 1.16). Due to baseline gender matching, there were 18 (36.0%) male pairs and 32 (64.0%) female pairs. Table 2 shows that the two groups were also aged matched. Majority of the students in both groups were from monogamous homes, and most had parents who were currently married. Most of their parents had secondary or higher level of education. The two groups were similar in all sociodemographic characteristics (Table 2). Also there were no statistically significant differences between the two groups’ scores in all baseline outcome measures (Table 3).

**Table 3:** Comparisons between intervention group and control group on outcome measures

Variables	Intervention group (n = 25 at baseline) (n = 21 at post intervention) *M(SD)*			Control group (n = 25 at baseline) (n = 16 at post intervention) *M(SD)*			*F* value	*P* value	np^2^
	Baseline	Post	Difference	Baseline	Post	Difference			
ISI	18.29 (1.22)^a^	6.76 (1.81)	11.53	13.25 (2.23)^g^	11.29 (2.10)	1.96	F (1, 34) = 1.10	0.0001	0.59
SOL (min)	15.65 (3.10)^b^	8.04 (2.79)	7.61	12.84 (3.26)^h^	19.88 (2.76)	− 7.04	F (1, 33) = 1.41	0.0001	0.39
TSD (h)	6.02 (1.54)^c^	8.43 (1.20)	2.41	5.78 (1.31)^i^	5.71 (1.46)	0.07	F (1, 33) = 1.03	0.0001	0.47
SMFQ	9.35 (1.64)^d^	3.55 (2.59)	5.8	8.42 (1.64)^j^	6.85 (2.39)	1.57	F (1, 31) = 1.32	0.002	0.34
SHQ	2.94 (1.67)^e^	2.54 (1.67)	0.4	2.80 (1.71)^k^	2.90 (1.64)	− 0.1	F (1, 32) = 1.08	0.08	0.15
KSQ	11.90 (1.23)^f^	16.82 (1.14)	4.92	12.05 (1.29)^l^	12.53 (1.31)	0.48	F (1, 34) = 1.02	0.001	0.36

T tests for between group comparisons at baseline and post intervention

*ISI* Insomnia Severity Index, *SOL* sleep onset latency, *TSD* total sleep duration, *SMFQ* Short Mood and Feelings Questionnaire, *SHQ* Sleep Hygiene Questionnaire, *KSQ* Knowledge of Sleep Questionnaire

^a^t (df) = 1.96 (48); p = 0.06

^b^t (df) = 0.61 (48); p = 0.55

^c^t (df) = 0.41 (48); p = 0.68

^d^t (df) = 0.51 (48); p = 0.61

^e^t (df) = 0.31 (45); p = 0.76

^f^t (df) = − 0.21 (48); p = 0.85

^g^t (df) = − 2.35 (35); p = 0.03

^h^t (df) = − 2.62 (34); p = 0.01

^I^t (df) = 4.11 (34); p = 0.0001

^j^t (df) = − 2.08 (32); p = 0.05

^k^t (df) = − 0.83 (35); p = 0.41

^l^t (df) = 4.40 (35); p = 0.0001

### Effect of the intervention on outcome measures

Between-group comparisons of post-intervention outcome measures (Table 3) showed that, compared with the control group, the intervention group had significantly lower insomnia {ISI; Mean (SD) 6.76 (1.81) vs. 11.29 (2.10), t = − 2.35, df 35, p = 0.03}, shorter sleep onset latency {SOL; Mean, (SD) 8.04 (2.79) vs. 19.88 (2.76), t = − 2.62, df 34, p = 0.01}, higher total sleep duration {TDS; Mean, (SD) 8.43 (1.20) vs. 5.71 (1.46), t = 4.11, df 34, p = 0.0001}, lower depressive symptoms {SMFQ, Mean (SD) 3.55 (2.59) vs. 6.85 (2.39), t = − 2.08, df 32, p = 0.05)}, and higher knowledge about sleep {Mean (SD) 16.82 (1.14) vs. 12.53 (1.31), t = 4.40, df 35, p = 0.0001}. However, there was not statistically significant difference in sleep hygiene {SHQ, Mean (SD) 2.54 (1.67) vs. 2.90 (1.64), t = − 0.83, df 35, p = 0.41)}.

Further multivariate analysis with ANCOVA showed that after controlling for baseline scores, the intervention showed statistically significant treatment effects on: reducing insomnia [ISI {F (1, 34) = 1.10, p = 0.0001, (ηp^2^ = 0.59}], reducing SOL {F (1, 33) = 1.41, p = 0.0001, ηp^2^ = 0.39}, increasing TSD {F (1, 33) = 1.03, p = 0.0001, ηp^2^ = 0.47}, reducing depressive symptoms [SMFQ {F (1, 31) = 1.32, p = 0.002 (ηp^2^ = 0.34}], and improving knowledge about sleep {F (1, 34) = 1.02, p = 0.001, ηp^2^ = 0.36}. However, the intervention had no significant treatment effect on sleep hygiene [SHQ {F (1, 32) = 1.08, p = 0.08 (ηp^2^ = 0.15}].

There was a statistically significant moderate negative correlation between the pre-post change ISI scores and Knowledge about sleep [(r (35) = − 0.36, p = 0.03].

### Participants’ feedback

All participants in the intervention group rated the programme as excellent (85.7%) or good (14.3%), and all would recommend it to others. They identified what they liked most about the intervention as; psycho-education about sleep physiology and sleep hygiene 42.9%), and relaxation techniques (28.6%). Their suggestions to improve the intervention included increasing the duration (44.4%), and expanding the programme to reach more adolescents (22.2%).

## Discussion

This five-session CBT-based intervention significantly reduced insomnia among the adolescents. Also, the treatment groups’ SOL, TSD, depressive symptoms, and knowledge about sleep all improved with large effective sizes. The intervention was well received by the adolescents. To our knowledge, this is the first published trial of a CBT-based intervention for adolescents with insomnia in Africa. Given that recent systematic reviews have highlighted the absence of similar studies in the region [9], the current study has made a contribution to the diversity and inclusivity of the literature on this subject.

Our results are in line with findings of other CBT-based trials among adolescents with insomnia as summarised in recent systematic reviews and meta-analysis [4–6, 9]. The previous studies show similar improvements in sleep and mental health outcomes with medium to large effect sizes. A specific contribution of the current study is to provide encouraging evidence that an adapted and contextualised CBT-based intervention for adolescent insomnia may also be effective and acceptable in African settings. Adequate sleep is crucial for normal adolescent development [9]. Therefore, finding effective psychological interventions for young people with disrupted sleep is important in all regions of the world.

The significant reduction in depressive symptoms is an important added benefit of CBT-based intervention in the current study. This finding is consistent with the results of meta-analysis [5]. The relationship between sleep and depression could be reciprocal in that, disrupted sleep is a feature of depression, while lack of restorative sleep may be a causal factor for depression [34]. The latter association may explain why psychological intervention for sleep difficulties is associated with reduction in depressive symptoms.

Some aspects of this study are worth highlighting due to their specific implications in the context of Nigeria and similar LMICs. The delivery of the intervention in school and during school hours increased accessibility as the adolescents did not need to make separate journeys to another location to receive the treatment. We made the sessions brief to fit within the school break-periods, so that participants did not have to miss school lessons. However, even with these measures aimed at improving access, only 28% of participants attended all 5 sessions which suggests that offering the intervention at a separate location requiring additional travelling might not have been viable. We adopted a simple cognitive strategy (count your blessings), which had shown acceptance and benefit in previous CBT-based interventions in the country [18, 19]. We nuanced and contextualised aspects of typical CBT-I such as stimulus control that did not fit the socio-ecology of the setting. Furthermore, the intervention was successfully delivered by a mental health professional with no prior specialist certification in CBT-I. In relation to the study’s aim to test reliability of outcome measures, we found that the ISI, which had not been used among Nigerian adolescents, was reliable in identifying adolescents with sleep difficulties and in detecting treatment effect. This finding makes the ISI a promising easy-to-complete outcome measure for future trials of insomnia treatment for adolescents in the region. Conversely, our locally derived measure of sleep hygiene showed limited reliability; hence requires further adaptation.

It is essential to evaluate the role of individual components of the type of eclectic interventions used in this study. Psycho-education is a key component of effective psychological interventions for insomnia [13], and it constituted a significant proportion of the manual used in the current study. We found a statistically significant moderate negative correlation between the pre-post change scores for insomnia and knowledge of sleep. This association suggests an important role for psycho-education in the current study. Also, psycho-education was the component that the participants liked most. This is not surprising, as the adolescents might have viewed the psycho-education sessions as providing them with practical information to help them with activities such as sleep.

The study has a number of limitations. Lack of technical and material resources in the region meant that we could not use actigraphy to obtain objective sleep data. This means that factors such as socially desirable responses within the intervention group’s self-report measures may have contributed to some of the positive findings. However, socially desirable responding could not explain the significantly improved knowledge of sleep by the intervention group compared with the control group. We made an active decision not to seek completion of sleep logs in order to reduce the burden of participation and improve feasibility of the study. Although small, the sample size was still adequate to detect the hypothesised treatment effects in this pilot study—even despite some losses due to Covid-19-related disruption. However, this limits the generalisability of the findings. Intent to Treat analysis would have been preferred but the disproportionate Covid-19-related loss of post-intervention data in the control group meant that Per Protocol analysis was more conservative and preferable. Finally, the single post-treatment data point means that sustainability of the treatment gains is unclear.

In conclusion, this study adds the first evidence from an African setting to the body of research supporting the effectiveness of CBT-based intervention in the treatment of adolescents with sleep difficulties. Larger clinical trials are required to further explore the efficacy and cost effectiveness of CBT-based intervention in the region. If resources permit, future studies should include objective actigraphy measured outcomes, and extended follow-up to assess sustainability of any treatment gains. There is an urgent need to establish contextualised, effective, accessible, and easily deliverable psychological interventions for adolescent insomnia in Nigeria and other African settings. Otherwise, millions of adolescents in the region will remain at risk of avoidable suffering from sleep difficulties.

## Data Availability

The datasets from the study are not publicly available because publication of raw data was not included in the consent forms. However, the data are available from the corresponding author on reasonable request.

## Abbreviations

ADHD: Attention Deficit and Hyperactivity Disorder
ANCOVA: Analysis of covariance
CBT: Cognitive Behavioural Therapy
CBT-I: Cognitive Behavioural Therapy for Insomnia
COVID-19: Corona Virus 19
HICs: High Income Countries
ISI: Insomnia Severity Index
LMICs: Low and Middle Income Countries
SES: Socio-economic status
SMFQ: Short Mood and Feelings Questionnaire
SHQ: Sleep Hygiene Questionnaire
SKQ: Sleep Knowledge Questionnaire
SOL: Sleep onset latency
TSD: Total sleep duration
WHO: World Health Organisation
